# The Methyl-CpG Binding Proteins Mecp2, Mbd2 and Kaiso Are Dispensable for Mouse Embryogenesis, but Play a Redundant Function in Neural Differentiation

**DOI:** 10.1371/journal.pone.0004315

**Published:** 2009-01-29

**Authors:** Isabel Martín Caballero, Janne Hansen, Donna Leaford, Steven Pollard, Brian D. Hendrich

**Affiliations:** 1 Institute for Stem Cell Research, MRC Centre for Regenerative Medicine, School of Biological Sciences, University of Edinburgh, Edinburgh, United Kingdom; 2 Wellcome Trust Centre for Stem Cell Research and Department of Biochemistry, University of Cambridge, Cambridge, United Kingdom; University of Washington, United States of America

## Abstract

**Background:**

The precise molecular changes that occur when a neural stem (NS) cell switches from a programme of self-renewal to commit towards a specific lineage are not currently well understood. However it is clear that control of gene expression plays an important role in this process. DNA methylation, a mark of transcriptionally silent chromatin, has similarly been shown to play important roles in neural cell fate commitment in vivo. While DNA methylation is known to play important roles in neural specification during embryonic development, no such role has been shown for any of the methyl-CpG binding proteins (Mecps) in mice.

**Methodology/Principal Findings:**

To explore the role of DNA methylation in neural cell fate decisions, we have investigated the function of Mecps in mouse development and in neural stem cell derivation, maintenance, and differentiation. In order to test whether the absence of phenotype in singly-mutant animals could be due to functional redundancy between Mecps, we created mice and neural stem cells simultaneously lacking *Mecp2*, *Mbd2* and *Zbtb33*. No evidence for functional redundancy between these genes in embryonic development or in the derivation or maintenance of neural stem cells in culture was detectable. However evidence for a defect in neuronal commitment of triple knockout NS cells was found.

**Conclusions/Significance:**

Although DNA methylation is indispensable for mammalian embryonic development, we show that simultaneous deficiency of three methyl-CpG binding proteins genes is compatible with apparently normal mouse embryogenesis. Nevertheless, we provide genetic evidence for redundancy of function between methyl-CpG binding proteins in postnatal mice.

## Introduction

DNA methylation is essential for mammalian development. Mouse embryos lacking either the de novo DNA methyltransferases (Dnmt3a and Dnmt3b) or the maintenance DNA methyltransferase (Dnmt1) die shortly after gastrulation [1], [2]. DNA methylation in mammals is predominantly associated with gene silencing, and DNA methylation-deficient cells and embryos show inappropriate expression of wide variety of genes [2]–[6]. The transcriptional silencing effect of DNA methylation is mediated at least in part through the action of the methyl-CpG binding proteins (Mecps). Mecps bind to methylated DNA and recruit chromatin remodelling proteins to facilitate transcriptional silencing (reviewed in [7]). Specific recognition of methylated DNA is mediated either via a methyl-CpG binding domain (MBD) or C2H2 zinc fingers. The MBD-containing Mecps include Mbd1, Mbd2, Mbd4 and Mecp2 [8]–[10], while Kaiso and the recently described ZBTB4 and ZBTB38 proteins use zinc fingers to bind methylated DNA [11], [12]. Both Kaiso and Mbd1 have the ability to bind unmethylated DNA as well as methylated DNA [13], [14], while Mbd4 preferentially binds G-T mismatches at methylated CpG sites [15]. Mecps have been shown to be associated with various methylated genes *in vivo*, however Mecp-dependent gene silencing has been shown for only a subset of these genes [16]–[21].

Targeted mutagenesis in mice has been used to study the functions of Mecps *in vivo*. Despite being widely expressed in mice, none of the Mecps assayed thus far is required for embryogenesis. Mice lacking Mbd1 are viable and fertile, displaying no overt phenotypes, although further investigation revealed that *Mbd1^(−/−)^* adult neural stem cells showed a defect in neuronal differentiation [22]. Mice lacking Mbd2 are similarly viable and fertile, but show abnormal maternal behaviour [23] and defects in T-cell development [20]. Mice deficient for *Zbtb33*, the gene which encodes the Kaiso protein, display no overt phenotypes [24]. In contrast, Mecp2-deficient mice display severe neurological defects and die at an average of six weeks of age [25], [26], while mutations in the human *MECP2* gene cause a range of neurological disorders, the most prevalent of which is Rett syndrome [27], [28].

Of all the Mecps for which functional data exists, only Mbd1 has been shown to play a role in neuronal differentiation. However it is notable that this function was shown for adult neural progenitors, but embryonic neurogenesis occurred normally in the absence of Mbd1 [22]. Mecp2 was specifically shown to function in neuronal maturation, not differentiation [29], [30], and while Mbd2 is implicated in controlling some aspects of behaviour, it has not been shown to play any role in neurons or neural development [23]. Despite the finding that *Zbtb33*-null mice show no neuronal phenotypes [24], the observation that Kaiso is highly expressed in brain [31] and was reported to be a component of the N-CoR co-repressor complex in HeLa cells [32] is suggestive of some neural function for this protein.

Although methyl-CpG binding proteins are expressed at varying levels in a wide variety of tissues and corresponding cDNAs can be detected in embryonic libraries, there is currently no evidence that mammalian methyl-CpG binding proteins play any role in embryonic development. This is in stark contrast to the situation in *Xenopus leavis*, where both xMecp2 and xKaiso are essential for normal embryogenesis [33]–[35]. This apparent discrepancy could be due to a significant difference between amphibians and mammals in the function of Mecps: mammalian Mecps might serve exclusively or predominantly redundant roles in embryogenesis, while amphibian Mecps each have distinct functions. Alternately mammalian Mecps may play no essential role during embryogenesis, but function only postnatally. In an attempt to distinguish between these two possibilities, we created mice and neural stem cells simultaneously lacking the three well-characterised methyl-CpG binding proteins not implicated in neuronal differentiation, namely Mbd2, Mecp2 and Kaiso. While we found no evidence for functional redundancy between these proteins in embryonic development, we do find evidence for overlapping functions in postnatal animals and in neural specification of neural stem cells in culture. Our findings strongly support the notion that in mammals, unlike in amphibians, methyl-CpG binding proteins are dispensable for embryonic development, but perform overlapping functions during neural development.

## Results

### Mbd2, Mecp2 and Kaiso are not required for mouse embryogenesis

**Figure 1:**
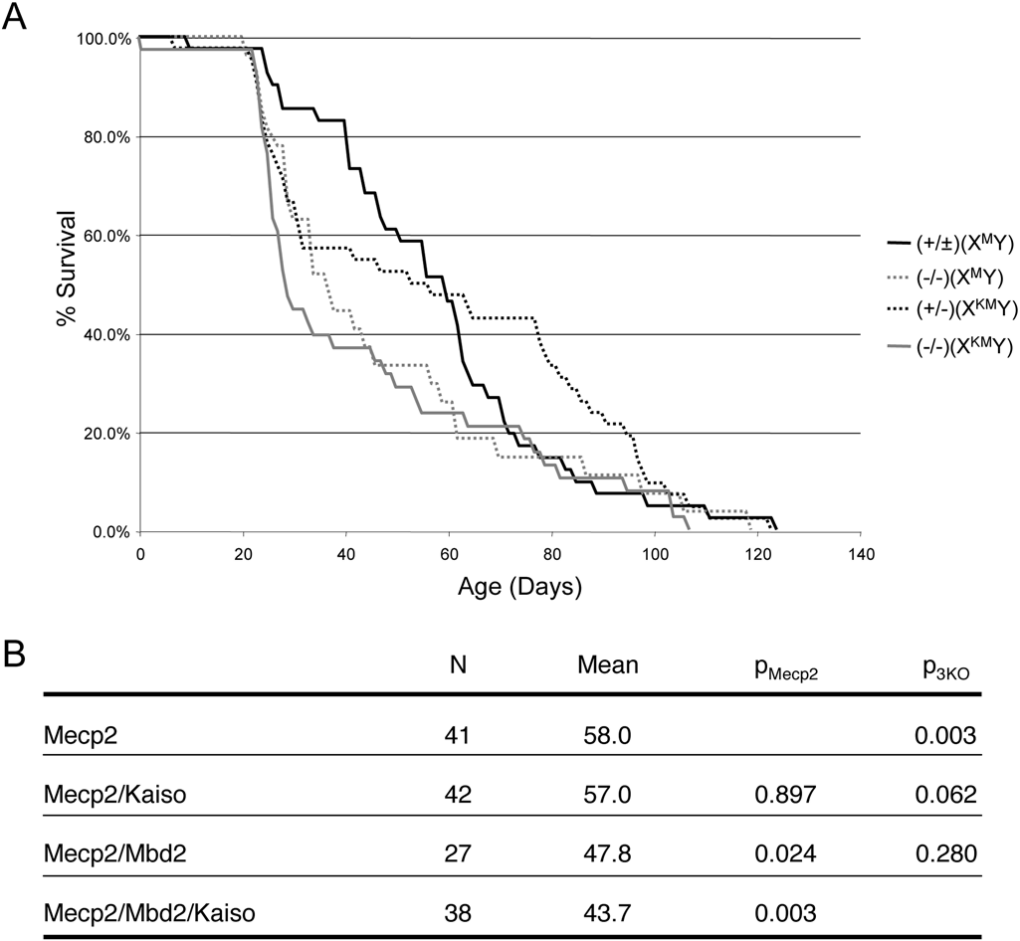
Genetic interaction between Mecps in postnatal animals. A. Cumulative plot of percentage of Mecp2-null mice (solid black line), Mecp2/Kaiso-double null mice (dotted black line), Mecp2/Mbd2-double null mice (dotted grey line) and Mecp2/Mbd2/Kaiso-triple null mice (solid grey line) surviving over time. All mice used in this analysis were produced from mouse lines that had undergone at least 6 generations of backcrossing to C57Bl/6 mice. B. Statistical analysis of the data pictured in A. N refers to the number of mice in the sample. A two-tailed Mann-Whitney test (http://faculty.vassar.edu/lowry/utest.html) was used to calculate p-values. p_Mecp2_ gives the p-value as compared to the Mecp2 single-null data, and p_3KO_ gives the p-value as compared to the triple null data.

To determine whether Mecps play a functionally redundant role during embryonic development, mice harbouring mutations for *Mbd2* [23], *Mecp2* [26] and *Zbtb33* [24] were intercrossed to make triple mutant animals. *Mecp2* and *Zbtb33* are both located on the X chromosome, while *Mbd2* is autosomal. In order to breed triple knockout (3KO) mice, *Mbd2^(−/−)^ or Mbd2^(+/−)^* females carrying the *Zbtb33 (Kaiso)* and *Mecp2* null alleles on the same X chromosome (designated “X^KM^X” in Table 1) were mated to *Mbd2*-null males. All expected combinations of single, double and triple mutant mice were viable and obtained at near-Mendellian frequencies (Table 1). Consistent with previous reports, *Mbd2* and *Zbtb33*-single null animals displayed no obvious phenotypes [23], [24]. *Mbd2*/*Zbtb33*-double null animals also showed no overt phenotypes. All mice carrying the *Mecp2*-null allele developed the characteristic Rett-like symptoms described previously [25], [26], and the presence or absence of *Mbd2* or *Zbtb33*genes did not alter the nature of the observable symptoms displayed by these mice, as previously reported [26]. Symptoms observed included tendency to have overgrown teeth, tremors, feeding, breathing irregularities and general mobility. No biometric tests were performed on these animals, so it is formally possible that the triple mutation resulted in some subtle changes in behaviour as compared to the single *Mecp2* mutation. In contrast, the average age of death for *Mecp2*-null mice did vary depending upon the mutational status of the other genes (Figure 1A). While *Mecp2*-null mice died at an average of 58 days, *Mecp2*-null mice also lacking the *Mbd2* gene or both the *Mbd2* and *Zbtb33*genes died significantly earlier, averaging 48 days (p<0.05) or 44 days (p<0.005), respectively (Figure 1B). *Mbd2^(−/−)^* mothers are known to display a nurturing phenotype [23], but the vast majority of double and triple null mice died after weaning and no correlation was found between age of death and maternal genotype at the *Mbd2* locus (Figure 1A and data not shown). Thus these genetic data are consistent with functional redundancy existing between methyl-CpG binding proteins in postnatal animals.

**Table 1:** Generation of triple KO mice.

Genotypes^a^	(+/−)(X^KM^X)×(−/−)(XY)^b^	(−/−)(X^KM^X)×(−/−)(XY)^b^
*Mbd2(+/−)Zbtb33(+/y)Mecp2(+/y)*	27	
*Mbd2(−/−)Zbtb33(+/y)Mecp2(+/y)*	21	7
*Mbd2(+/−)Zbtb33(−/y)Mecp2(−/y)*	23	
*Mbd2(−/−)Zbtb33(−/y)Mecp2(−/y)*	18	5
*Mbd2(+/−)Zbtb33(−/y)Mecp2(+/y)* c	4	
*Mbd2(−/−)Zbtb33(−/y)Mecp2(+/y)* c	6	1
*Mbd2(+/−)Zbtb33(+/y)Mecp2(−/y)* c	2	
*Mbd2(−/−)Zbtb33(+/y)Mecp2(−/y)* c	5	2

a Genotypes of male mice at weaning or death.

b Genotypes of parents crossed to produce listed progeny. “(+/−)” and “(−/−)” refer to the *Mbd2* genotype, while “X^KM^” indicates an X chromosome harbouring null alleles at both *Zbtb33* (*Kaiso*) and *Mecp2*.

c Genotypes arising from nondisjunction between the *Zbtb33* and *Mecp2* loci during maternal meiosis.

### Methyl-CpG binding proteins are expressed in neural cell types

**Figure 2:**
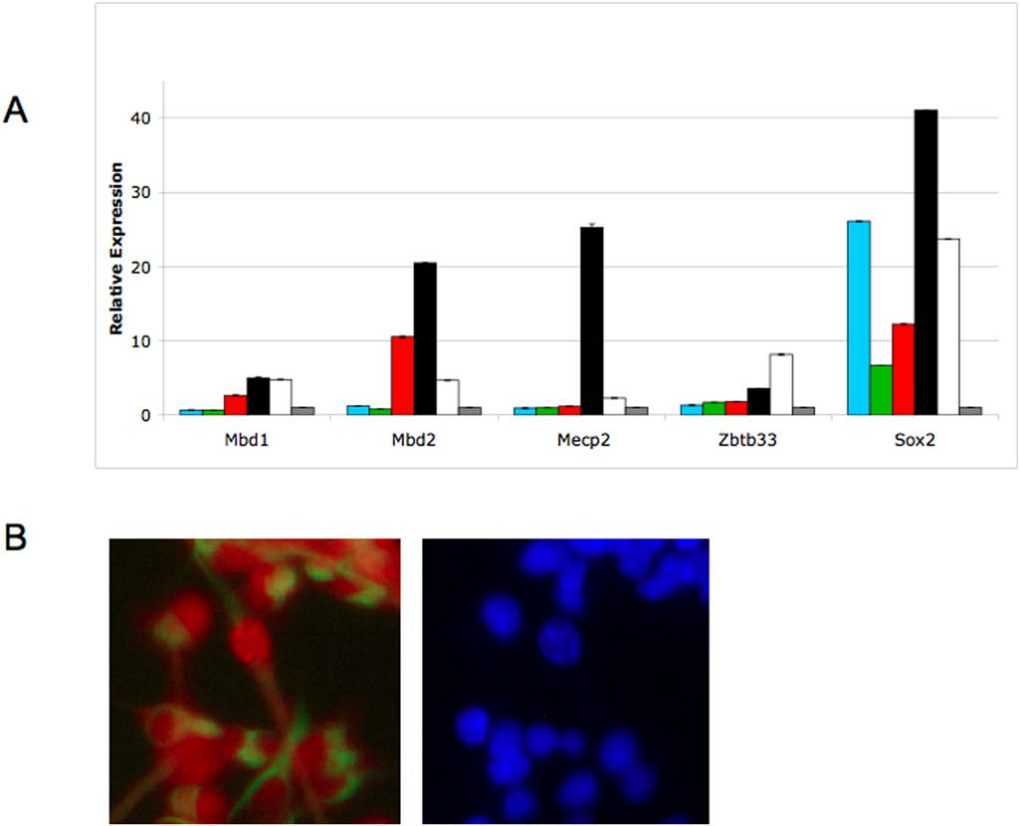
Methyl-CpG binding proteins in NS cells. Expression of *Mbd1*, *Mbd2*, *Mecp2*, *Zbtb33* and *Sox2* detected by RT-PCR in ES cells (blue boxes), FACS-purified, Sox1-expressing ES-derived neural progenitors (green boxes), NS cells (red boxes), NS cell -derived neuronal cultures (black boxes), NS cell -derived astrocyte cultures (white boxes), and whole brain (grey boxes). Error bars indicate SEM. For each cell sample, expression was normalized to *Gapdh* expression, and is shown relative to that seen in adult mouse brain. B. Detection of Mecp2 protein in NS cells. Mecp2 can be detected by antibody staining (red) in NS cell cultures, also expressing the NS cell marker RC2 (green). The Dapi-stained image is shown at right.

In order to determine whether Mecp activity is detectable in neural cell types, expression levels of methyl-CpG binding protein genes were measured quantitatively in embryonic stem (ES) cells and their differentiated progeny (Sox1-expressing neuroepithelial cells, neural stem cells and Gfap-expressing astrocytes; see Materials and Methods for details). *Sox2* expression was used as a control, as its expression pattern is well characterised in neuronal development [36], [37]. As expected, *Sox2* is well expressed in ES cells, NS cells, and neurons (Figure 2A). All cells present in the astrocyte-like cultures express the astrocyte marker Gfap (e.g. [38] and see below), although after three days in differentiation conditions they still show appreciable expression of NS cell markers (e.g. *Sox2* and *Nestin*; Figure 2A). Neural cultures contain a heterogeneous mix of cells including NS cells, neurons and astrocytes [38]. Expression of all four methyl-CpG binding protein genes was detectable in each of the cell types used in this study. *Mecp2* expression was highest in neuronal cultures, consistent with its published roles in neuronal maturation, but not differentiation [29], [30]. *Mbd1*, the only Mecp for which a function in NS cells has been reported [22], was well expressed in NS cells and neurons. *Mbd2* is well expressed in NS cells and neurons, and while *Zbtb33* was expressed only moderately in neuronal cultures, it showed highest expression in astrocyte-like cultures. Nevertheless expression of all methyl-CpG binding protein genes was detectable in NS cells (Figure 2A, B).

### Mbd2, Mecp2 and Kaiso are dispensable for neurosphere formation and potency

**Figure 3:**
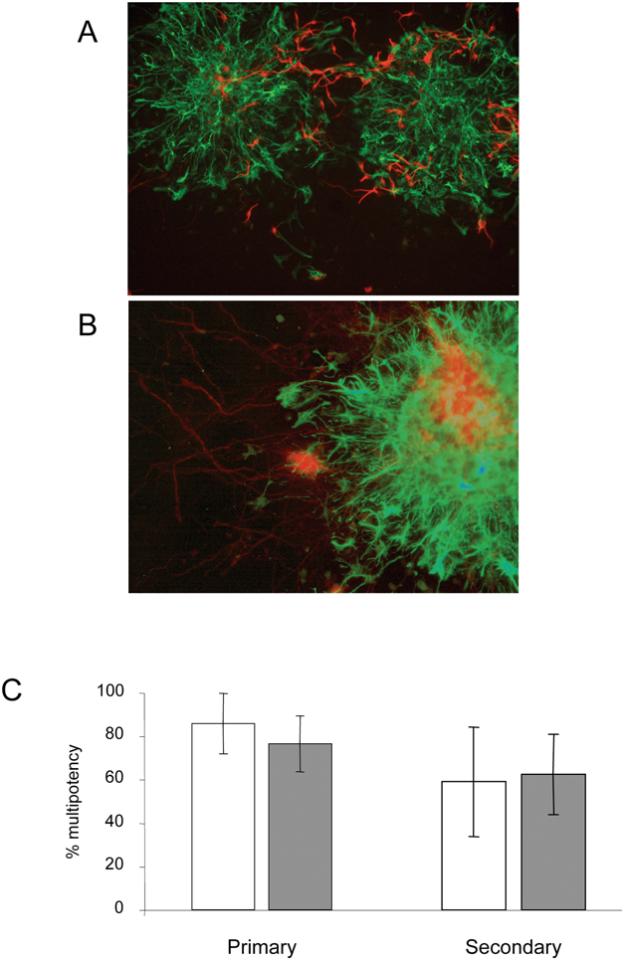
Neurosphere potency. Wild-type (A) or 3KO (B) neurospheres made from E14.5 cortex were differentiated for five days and stained for ß-tubulin III (red) and Gfap (green). C. Proportion of colonies formed staining for both neural (ß-tubulin III) and astrocyte (Gfap) markers when primary or secondary neurosphere cultures were allowed to differentiate. Data for wild-type neurospheres are shown in open boxes; that for 3KO neurospheres is shown in filled boxes. Error bars represent SEM.

DNA methylation has been shown to restrict astroglial differentiation in the developing mouse brain [39]. If this function of DNA methylation is mediated by Mecps, we would expect to see precocious or preferential astroglial differentiation of Mecp-deficient neurospheres. In order to test this possibility, neurospheres were made from both wild-type embryos and from 3KO embryos at embryonic day 14.5. We saw no obvious difference in the formation, frequency or size of neurospheres made from wild-type and 3KO embryos (data not shown). Neurospheres were cultivated for one week and then plated out in adherent culture in differentiation medium to assess their differentiation potential. In order to test the ability of neurospheres to self-renew, primary neurospheres were dissociated to single cells and allowed to form secondary neurospheres prior to plating out in differentiation conditions. We found that primary and secondary neurospheres derived from 3KO embryos showed a similar degree of formation, frequency, size (data not shown) and potential to form neurons and astrocytes as those formed from wild-type embryos (Figure 3). This result indicates that Mbd2, Mecp2 and Kaiso are not important for the DNA methylation-mediated inhibition of astroglial differentiation in neurospheres.

### Derivation and differentiation of 3KO neural stem cell lines

**Figure 4:**
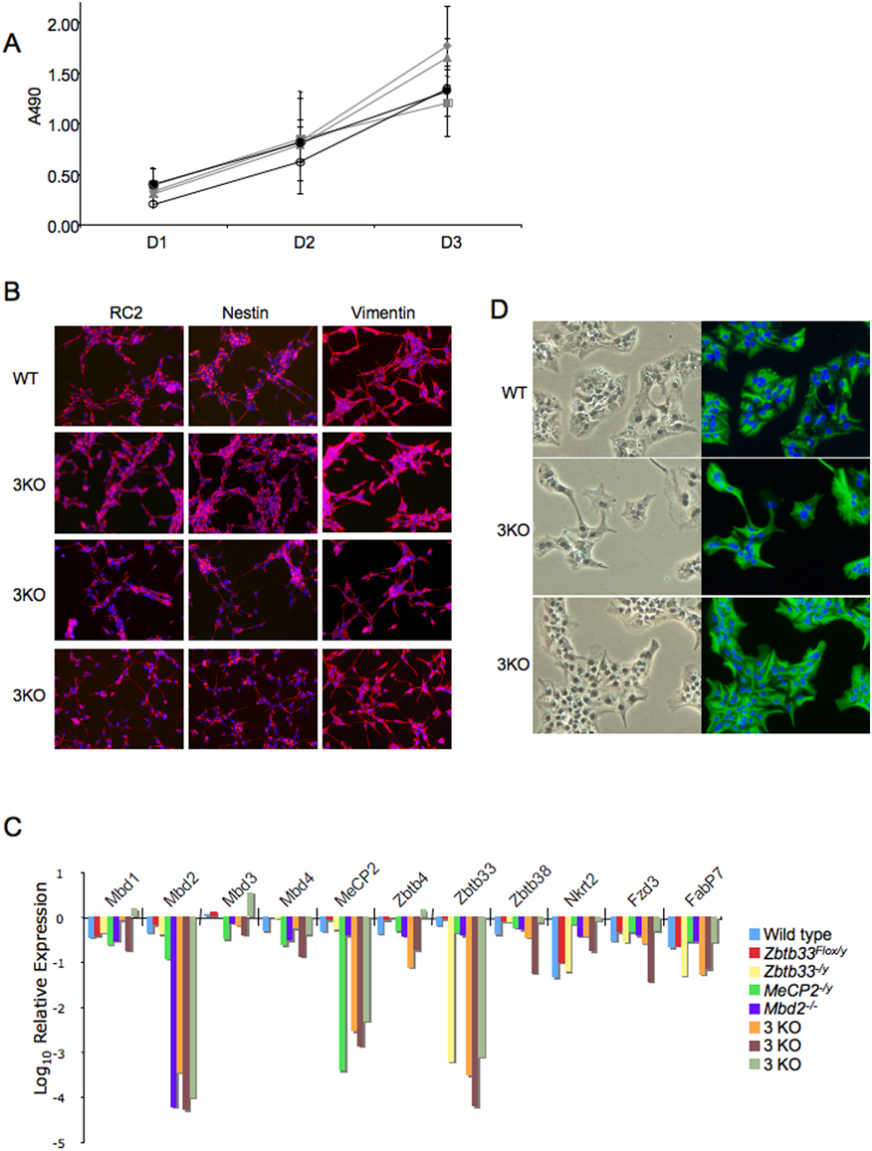
3KO Neural stem cells. A. Proliferation rates of three 3KO NS cell lines (grey lines) and two wild-type NS cell lines (black lines) were measured by the MTT assay over three days. Error bars represent SEM. B. Immunocytochemistry of the indicated NS cell markers (red staining) in one wild-type NS cell line (top panels) and three independent 3KO NS cell lines. Cells were counterstained with Dapi (blue). C. Expression levels of the genes indicated across the X-axis were measured in wild type, single mutant, and triple mutant NS cell lines (as indicated in the legend). Expression was monitored in triplicate in two biological replicates by quantitative PCR, and results of a representative experiment is shown. Expression levels were normalized to Sox2 expression and are shown relative to the levels found in an E14.5 cortex-derived wildtype NS cell line. The *Zbtb33^−/y^* NS line was made by Cre transfection of the *Zbtb33^Flox/y^* NS line, as described [24], while the remaining mutant NS lines were all derived from E14.5dpc embryos. D. Immunostaining of NS cell cultures after four days in the presence of serum and removal of growth factors to induce Gfap activation. Left panels: bright field, right panels: Gfap (green), Dapi (blue). Top panels: WT cells, middle and bottom panels: two independent 3KO cell lines.

Neurospheres have long been used to study neural stem cells, however they have the limitation of being composed of a heterogeneous mix of neural stem cells and differentiating progeny [40]. Therefore it was possible that any subtle effect of Mecp-deficiency on neural differentiation might not be detectable using this system. In contrast, neural stem (NS) cell lines [38] are a completely homogeneous stem cell population, which were subsequently used to more rigorously address whether Mecps play any role in neural differentiation. NS cell lines were made from both wild-type and 3KO 14.5dpc embryos. We could see no genotype-dependent differences in the efficiency or rate of establishment of NS cell lines. Similarly, no difference in proliferation rates could be detected between the 3KO and wild-type NS cells (Fig. 4A). Like wild-type NS cells, 3KO NS cells were able to form colonies from single cells after plating at clonal density (data not shown). Both wild-type and 3KO NS cell lines showed uniform expression of neural stem cell markers (RC2, nestin and vimentin) and an absence of differentiated cell markers (TuJ1 and Gfap) by immunofluorescence (Fig. 4B). To test whether the loss of three methyl-CpG binding proteins was compensated for by upregulation of other methyl-CpG binding proteins, expression of *Mbd1*, *Mbd2*, *Mbd3*, *Mbd4*, *Mecp2*, *Zbtb4*, *Zbtb33* and *Zbtb38* was monitored in three independently-derived 3KO NS lines, in all three of the single mutant NS lines and in three different wild-type NS lines (Figure 4C). This analysis showed no evidence for consistent up-regulation of any of the known methyl-CpG binding protein genes in 3KO NS cells. Therefore we conclude that Mbd2, Mecp2 and Kaiso are dispensable for NS cell derivation and maintenance.

**Figure 5:**
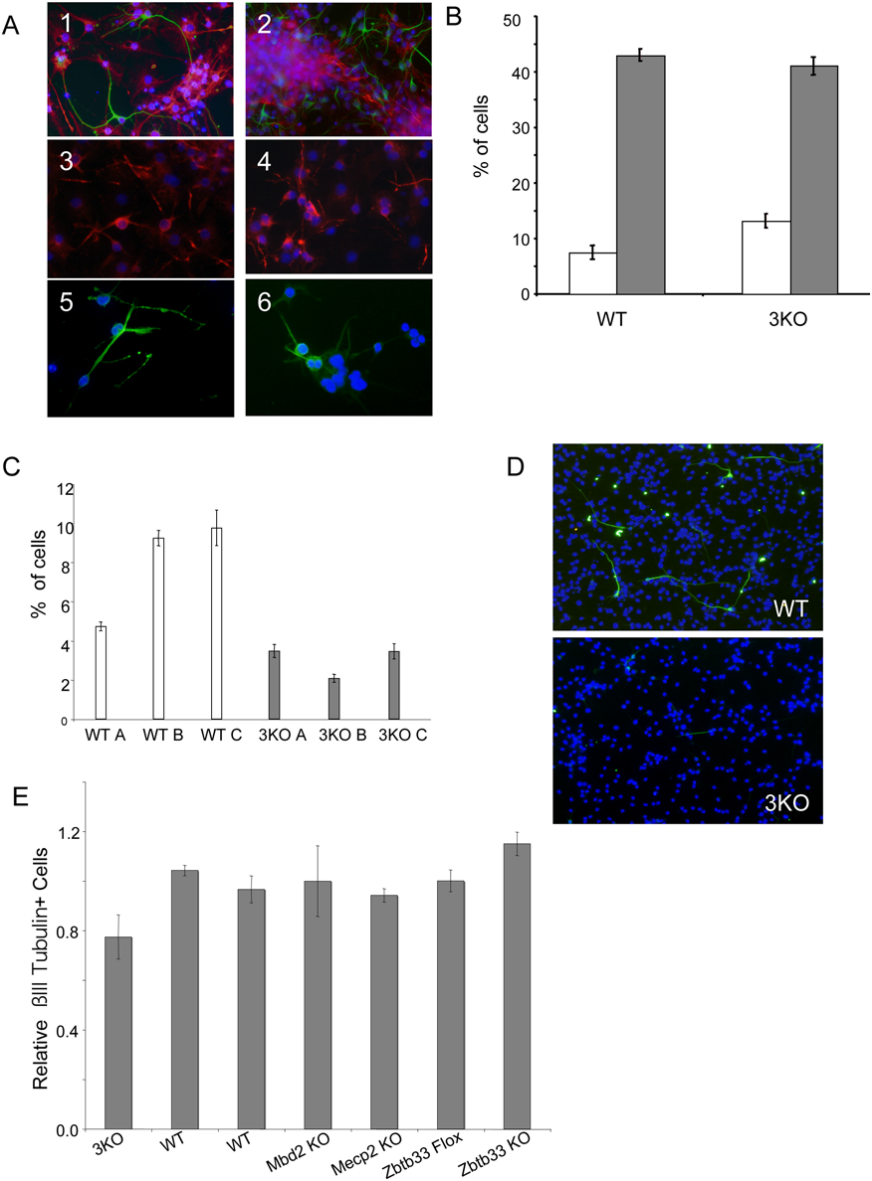
Neural differentiation of 3KO NS Cells. NS cells were cultured for 15 days in neuronal differentiation conditions and immunostained for ß-tubulin III (green) and Gfap (red) in panels 1 and 2, Map2 in panels 3 and 4, and for Gad67 (green), a marker of Gabaergic neurons, in panels 5 and 6. Cells were counterstained in Dapi (blue). Panels 1, 3, and 5: wild-type NS cells; panels 2, 4, and 6: 3KO NS cells. B. Quantitation of the number of ß-tubulinIII expressing (white bars) and Gfap-expressing (grey bars) cells in 14 day neural differentiation cultures from wild-type (left) and 3KO (right) NS cell cultures. Error bars represent SEM from three independent experiments. C. Quantitation of the number of ß-tubulinIII positive cells present in NS cell cultures after seven days' neuronal differentiation. Data is shown for three different wild-type and three different 3KO NS cell lines. Error bars represent SEM from at least three independent experiments. The difference seen between the wild-type and 3KO lines is highly statistically significant (p<0.0024, Bonferroni). D. Images of 7 day neural cultures from wild-type (left) or 3KO (right) NS cells, stained for ß-tubulin III (green) and Dapi (blue). E. Relative percentage of ß-tubulin III positive cells present in NS cell cultures of indicated genotype after eight days' neuronal differentiation. Error bars represent SEM from at least four independent fields of cells. All cell lines were derived from E14.5 cortex except those labeled “*Zbtb33* Flox” and “*Zbtb33* KO”, which are ES cell-derived NS lines, with the *Zbtb33* KO line having been derived from the *Zbtb33* Flox line by Cre-transfection and gene deletion. Hence data for the *Zbtb33* Flox line is included as an appropriate wild-type control for the *Zbtb33* -null NS line.

3KO NS cells were able to activate expression of Gfap in response to serum stimulation and produce cells displaying an astrocyte-like morphology, indistinguishable from wild-type cells (Figure 4D). We found no evidence for unscheduled or impaired astroglial differentiation of 3KO NS cell cultures (Figure 4D and data not shown). Nevertheless it remained possible that 3KO NS cells would show early and/or preferential activation of Gfap upon induction in culture. In order to test this possibility, activation of Gfap expression was monitored 8, 12, 20 and 24 hours after induction by serum stimulation in wild-type and 3KO NS cells. No genotype-dependent difference in the activation rate of Gfap was found between the different cell lines (data not shown). Neither could we identify any genotype-specific differentiation differences when varying passage number, cell density, serum concentration or presence/absence of LIF (data not shown). Similarly, when subjected to a neuronal differentiation protocol for two weeks 3KO NS cells were found to be capable of forming cells expressing neuronal or glial markers that displayed either neuronal or astrocytic morphologies, respectively (Figure 5A,B). Thus we conclude that Mbd2, Mecp2 and Kaiso are not required for formation of neurons or astrocytes from NS cells in culture.

No evidence for altered neurogenesis or gliogenesis in 3KO NS cell cultures was detected after two weeks of differentiation. However we did find evidence for Mecp function in initial neuronal specification. Our standard neuronal differentiation procedure involves the induction of neurogenesis through withdrawal of Egf for seven days, followed by a period of neural growth stimulated by FGF withdrawal and addition of B27 to the media for a further seven days [38]. When differentiation cultures were stained after Egf withdrawal, but prior to removal of FGF, we found that 3KO NS cultures showed significantly reduced number of Tuj1-positive cells displaying an immature neural morphology as compared to the wild-type cultures (Figure 5C). After a further day of differentiation the difference is reduced although still detectable (Figure 5E), but by 14 days of differentiation the 3KO NS cultures appear to have completely recovered from this initial defect as we could no longer detect a difference in the number of Tuj1-expressing cells (Figure 5B). Hence we conclude that while triple knockout NS cells show an initial impairment in neural differentiation, this is a transient effect and the cells manage to recover after a further week in culture.

To determine whether this observed defect in neural differentiation is due to any one of these *Mecp* genes, or is indicative of functional redundancy between them, we made NS cells lacking each gene individually, i.e. *Mecp2^−/y^* NS cells, *Mbd2^−/−^* NS cells, and *Zbtb33^−/y^* NS cells. Each line was subjected to the neural differentiation protocol and Tuj1-expressing cells were counted after 7 days. Each of the single mutant NS cell lines was found to produce a normal number of neurons in this assay (Figure 5E). Thus we conclude that the defect observed in triple knockout NS cells likely results from some redundant function between these three proteins.

## Discussion

In this study we show that we can find no evidence for functional redundancy between three methyl-CpG binding proteins, Mecp2, Mbd2 and Kaiso, in embryonic development or in the derivation or maintenance of neural stem cells in culture. Nevertheless, we do provide evidence for a role for these three proteins in differentiation of neural stem cells and in postnatal mice. Mice lacking three methyl-CpG binding proteins succumb to Mecp2-dependent effects significantly earlier than do *Mecp2* single null mice, while *Mbd2* and *Zbtb33* single or double-null mice show no reduced viability. Further we find an initial defect in neuronal specification of triple null NS cells after one week of differentiation. This manifests as delayed neuronal commitment, but not as altered fate choice as the these cells are able to overcome this initial defect and recover normal numbers of neuron-like cells after a further week in culture. While other studies have demonstrated a lack of redundant function for two or more Mecps in silencing of specific loci (e.g. [41]), this study provides the first evidence for a redundant function of Mecps in mammalian cells.

Interestingly, it has recently been reported that expression of a dominant negative Ntrk2 protein in embryonic cortical precursors results in an initial decrease in the rate of neurogenesis, but that cultures recover in the longer term to give normal differentiation rates [42], mirroring very closely the phenotype we report here. Mecp2 has previously been shown to maintain silencing of *Bdnf*, the gene encoding an Ntrk2 ligand [17], [43]. Tantalising as this potential link is, we did not see a consistent difference in the expression levels of *Ntrk2* in our triple knockout NS cell lines (Figure 4C).

We find no evidence for Mecp function during murine embryogenesis (Table 1). Given that DNA methylation plays a well documented role in neural cell fate specification [39], [44], [45] and is essential for mammalian embryogenesis [2], [5], our findings support the conclusion that interpretation of the DNA methylation mark by methyl-CpG binding proteins plays little if any role in these functions. It is formally possible that any essential, developmental functions of mammalian methyl-CpG binding proteins are being carried out by one or both of the Kaiso-like proteins Zbtb4 and Zbtb38 [12], and/or Mbd1. While we cannot rule this possibility out, as yet no developmental functions have been defined for Mbd1 [22], Zbtb4 or Zbtb38. Further, we did not see increased transcript levels of *Mbd1*, *Mbd4*, *Zbtb4* or *Zbtb38* in the multiple knockout NS cells (Figure 4C), making it unlikely that increased expression of one or more of these is compensating for loss of Mbd2, Mecp2 and Kaiso.

Despite a lack of evidence for a requirement for Mecps during embryogenesis, we found evidence for overlapping functions in postnatal animals (Figure 1). It is not clear why the 3KO mice succumb to Mecp2-dependent death earlier than do Mecp2 single-null mice. Given the similarity of symptoms displayed by all Mecp2-null animals in this study prior to death, irrespective of whether they also lack *Mbd2* and/or *Zbtb33*, it is likely that Mbd2, and possibly Kaiso, are able to compensate somewhat for the role Mecp2 normally plays in the postmitotic neurons of Mecp2-null mice, although phenotypic synergy between the different mutations cannot be completely ruled out. The difference in life span between *Mecp2*-single null and *Mbd2/Mecp2*-double null animals was not seen in a previous report [26] in which mice were on a mixed genetic background, whereas the mice in the current study were on a C57Bl/6 background, indicating the existence of strain-specific modifiers of life span in *Mecp2*-null mice. Using chromatin immunoprecipitation, Klose et al. found no evidence for co-localisation of MBD2 and MECP2 binding sites in a human lung cell line [46], consistent with the observation of tissue-specific functions for methyl-CpG binding proteins [22], [24]–[26], [47], and/or for cooperative regulation by different Mecps biding to distinct sites within a given gene.

Our finding that Mbd2, Mecp2 and Kaiso have no developmental role in mammals contrasts with the situation in *Xenopus laevis*, where depletion of xKaiso results in severe embryologic defects [33], [34] and xMecp2 is essential for normal neural development [35]. This discrepancy may indicate the existence of a significant difference in the use of DNA methylation and/or methyl-CpG binding proteins in amphibians and mammals. The Mbd3 protein provides evidence for a change in the use of methyl-CpG binding proteins during mammalian evolution [48]. The *Mbd3* gene found in eutherian genomes encodes a protein incapable of binding to methylated DNA due to point mutations within its MBD [49], while non-eutherian vertebrate genomes encode Mbd3 proteins containing an intact methyl-CpG binding domain [48]. In contrast to the Mecps which are not important for mammalian embryonic development, Mbd3 is essential for embryogenesis in both mice and frogs [23], [50].

Animal genomes underwent a significant change in DNA methylation patterns at the invertebrate-vertebrate boundary, which likely coincided with a change in the use of DNA methylation by those genomes [51]–[53]. Amplification and diversification of the Mecp family in vertebrates coincided with the spreading of DNA methylation across the genome, and likely contributes to a ‘fine tuning’ mechanism of gene expression [53], [54]. Given this evolutionary history, and the fact that no evidence exists for an essential, embryonic function for any mammalian methyl-CpG binding protein, we propose that the mammalian Mecps have evolved to function predominantly or exclusively to fine tune gene expression in postmitotic tissues. A formal proof of this theory, requiring the simultaneous deletion (or knock-down) of all known methyl-CpG binding transcriptional repressor proteins in mammalian embryogenesis, has yet to be achieved.

## Materials and Methods

### Animals

*Mbd2*-null [23], *Mecp2*-null [26] and *Zbtb33*-null [24] mice have been described. Animal work was approved by the appropriate ethics committees of the School of Biological Sciences, University of Edinburgh, and by the UK Home Office. Multply mutant mice were created by standard intercrossing. *Zbtb33* and *Mecp2* are located approximately 36 Mb apart on the X chromosome (http://genome.ucsc.edu/cgi-bin/hgGateway). To make mice doubly heterozygous or doubly hemizygous for these two alleles, females harbouring the *Zbtb33* deletion on one X chromosome and the *Mecp2* deletion on the other were crossed to wild type males, and any progeny inheriting both mutant alleles were judged to have received a recombined X chromosome containing both mutant alleles. Of 133 pups generated from such crosses, 11 were judged to contain the recombined chromosome, providing a recombination frequency of 8.3%.

### Neurospheres and Neural stem cell culture

Neurospheres were prepared as described [55]. Embryonic age E14.5 cortices were dissected and meninges removed. Cells were mechanically dissociated, passed through a 40 µm nylon mesh (BD Falcon) and suspended at a density of 5000/cm^2^ in 25 cm^2^ culture flasks in the presence of FGF (Prepotech) in DMEM/F12 supplemented with B27 and penicillin/streptomycin. After 7 days primary neurospheres were subjected to form secondary neurospheres by mechanical dissociation and prepared as explained above. For differentiation, primary and secondary neurospheres were plated on to poly-L-lysine-coated (Sigma) coverslips in the presence of Neurobasal media supplemented with B27, L-glutamine, 2-mercaptoethanol and penicillin/streptomycin for 5 days. Neural stem cell lines were made and maintained as described [38]. Briefly, embryonic age E14.5 cortices were dissected and dissociated as for neurospheres. The entire cell suspension from each embryonic cortex was plated on 25 cm^2^ flasks in NS media [38]. Cultures were allowed to form neurospheres for 7 days, after which they were collected by centrifugation and plated in gelatinised flasks. From this point cells were maintained as an adherent monolayer and passaged when 30–50% confluent. Passage number was always kept as low as possible to minimise the chances of genetic or epigenetic change in the cultures and will typically be less than p30. Astrocytic and neuronal differentiation was performed as described [37].

To generate Sox1-expressing neural precursors, *Sox1-GFP* knock-in ES cells were cultured in DMEM/F12 media (Invitrogen) supplemented with N2 and B27 (Invitrogen) as described [56]. Cultures were subjected to FACS purification of GFP-expressing cells at day 6 of differentiation, followed immediately by RNA extraction. ES with no GFP knock-in were similarly subjected to this monolayer differentiation procedure and used as a control to identify autofluorescence events.

### RT-PCR

Expression levels of methyl-CpG binding protein genes were measured quantitatively in embryonic stem (ES) cells, ES cell-derived, FACS sorted, Sox1-Gfp positive cells [56], ES cell-derived and E14.5 cortex-derived neural stem (NS) cell cultures [38], Gfap-expressing astrocyte-like cultures (day 3) and neuronal differentiation cultures (day 14) [38]. *Sox2* expression was used as a control, as its expression pattern is well characterised in neuronal development [36], [37]. All cells present in the astrocyte-like cultures express the astrocyte marker Gfap (e.g. Figure 4C), although after three days in differentiation conditions they still show appreciable expression of NS cell markers. Neural cultures contain a heterogeneous mix of neurons, astrocytes and NS cells as described [38] (e.g. Figure 5A). Primer sequences are in Table 2.

**Table 2:** RT-PCR Primers used in this study.

	Forward	Reverse
Mbd1	GAGCACAGAGAATCGCCTTC	CACACCCCACAGTCCTCTTT
Mbd2	CTGGCAAGATACCTGGGAAA	TTCCGGAGTCTCTGCTTGTT
Mbd3	AGAAGAACCCTGGTGTGTGG	TGTACCAGCTCCTCCTGCTT
Mbd4	ACAGGATGGCTCTGAAATGC	TCTACTTGTGTCCGTGGGATG
Zbtb33	GGAGCAGGCATGGAGAGTAG	GGAATTTTCGGTCTTCCACA
MeCP2	CAGGCAAAGCAGAAACATCA	GCAAGGTGGGGTCATCATAC
Zbtb4	GCTGCCTTCTCCGATGTCC	CAGTAGGATCTGAGGTGACCA
Zbtb38	CATCTTTTGGAGCCATACGATCT	TGACGGTTTCCTGTCTTTTGAC
Nkrt2	ATCAGCTATCGAACAATGAGG	TCAAGAGGTTCGTCGTGTAG
Sox2	GCGGAGTGGAAACTTTTGTC	TATTTATAATCCGGGTGCTCCTT
FabP7	CCAGCTGGGAGAAGAGTTTG	TTTCTTTGCCATCCCACTTC

### Immunofluorescence

Antibodies against Mecp2 (Millipore), Gfap (Santa Cruz Bioechnologies or Sigma), ß-tubulin III (Covance), Map2 (Sigma), Gad67 (Chemicon), RC2 (DSHB), Vimentin (DSHB), and Nestin (DSHB) were used as described below. Secondary antibodies were obtained from Invitrogen. Cells cultured in tissue culture plates were fixed with 4% PFA and washed three times in PBS. Cells were blocked in 3% serum and 0.1% Triton-X for 60 min. Primary antibodies were diluted in 3% serum PBS solution and incubated overnight at 4°C. Cells were washed three times in PBS and incubated in secondary antibody solution (3% serum in PBS) for 1 hour at room temperature. After this incubation time, secondary antibody solution was removed and cells were washed three times in PBS. Either Volocity software (Improvision) or manual counting was used for cell quantification.

### MTT assay

Cell proliferation was measured using the CellTiter^R^ 96 AQ_ueous_ one solution cell proliferation assay (Promega) according to manufacturer's instructions with the following modifications: 10×10^4^cells were plated in 150 µl of media and 30 µl of CellTiter reagent was added to the culture. The absorbance was read after 4 hours using a precision microplate reader (Molecular Devices) at 490 nm wavelength.
